# A novel toolbox for *E. coli* lysis monitoring

**DOI:** 10.1007/s00216-016-9907-z

**Published:** 2016-09-02

**Authors:** Vignesh Rajamanickam, David Wurm, Christoph Slouka, Christoph Herwig, Oliver Spadiut

**Affiliations:** 1 Research Division Biochemical Engineering, Institute of Chemical Engineering, Vienna University of Technology, Getreidemarkt 9/166, 1060 Vienna, Austria; 2 Christian Doppler Laboratory for Mechanistic and Physiological Methods for Improved Bioprocesses, Institute of Chemical Engineering, Vienna University of Technology, Gumpendorfer Strasse 1a, 1060 Vienna, Austria

**Keywords:** Bioprocess monitoring, Data science tools, Lysis, *Escherichia coli*, Chromatogram fingerprinting, HPLC

## Abstract

The bacterium *Escherichia coli* is a well-studied recombinant host organism with a plethora of applications in biotechnology. Highly valuable biopharmaceuticals, such as antibody fragments and growth factors, are currently being produced in *E. coli*. However, the high metabolic burden during recombinant protein production can lead to cell death, consequent lysis, and undesired product loss. Thus, fast and precise analyzers to monitor *E. coli* bioprocesses and to retrieve key process information, such as the optimal time point of harvest, are needed. However, such reliable monitoring tools are still scarce to date. In this study, we cultivated an *E. coli* strain producing a recombinant single-chain antibody fragment in the cytoplasm. In bioreactor cultivations, we purposely triggered cell lysis by pH ramps. We developed a novel toolbox using UV chromatograms as fingerprints and chemometric techniques to monitor these lysis events and used flow cytometry (FCM) as reference method to quantify viability offline. Summarizing, we were able to show that a novel toolbox comprising HPLC chromatogram fingerprinting and data science tools allowed the identification of *E. coli* lysis in a fast and reliable manner. We are convinced that this toolbox will not only facilitate *E. coli* bioprocess monitoring but will also allow enhanced process control in the future.

**Electronic supplementary material:**

The online version of this article (doi:10.1007/s00216-016-9907-z) contains supplementary material, which is available to authorized users.

## Introduction

*Escherichia coli* is one of the most popular host organisms for recombinant protein production (e.g., [1, 2]). However, strong induction of recombinant protein production results in great cell stress and high metabolic burden, potentially leading to cell death and lysis [3]. Therefore, it is of utmost importance to monitor the physiological state of the cells to minimize product loss. Flow cytometry (FCM) is the predominant method to monitor and quantify *E. coli* cell death. However, FCM devices are expensive and therefore often not available. Furthermore, FCM measurements need manual intervention and often require time-consuming, offline sample preparation. In contrast, spectroscopic methods, such as RAMAN and near infrared spectroscopy (NIR), can be used for online monitoring [4, 5]. Owing to the high magnitude of multi-dimensional data derived from these methods, multivariate data analysis (MVDA) is used for data interpretation [6, 7]. However, the continuously changing media background, changing morphologies, as well as changing process parameters (e.g., aeration) cause inaccuracy in measurements and thus limit the applications of these methods. Thus, alternative strategies for bioprocess monitoring are needed.

In this study, we developed a novel toolbox based on UV chromatograms as fingerprints to identify *E. coli* cell lysis. To date, UV spectroscopy coupled to high pressure liquid chromatography (HPLC) is implemented for real-time monitoring in downstream processes [8]. However, we hypothesized that UV chromatographic data of *E. coli* bioprocess samples contain information about impurity release and lysis events and thus can also be used in upstream processing. We followed the impurity pattern of nucleic acids at 260 nm as marker for cell lysis along different *E. coli* bioprocesses. We combined UV chromatographic data with chemometric methods to identify lysis which may be used to define the optimal time point of harvest.

## Materials and methods

### Strain

*E. coli* BL21 (DE3) (Life technologies, CA, USA) and the pET28a(+) expression vector were used for the production of the cytoplasmic recombinant single-chain antibody fragment (scFv).

### Bioreactor cultivations

In all cultivations, a minimal medium according to DeLisa [9] supplemented with 0.02 g/L Kanamycin was used. Three cultivations were carried out in a DASGIP multi bioreactor system with four glass bioreactors and a working volume of 2 L each (Eppendorf, Germany). Detailed information about this fermenter setup can be found elsewhere [10].

An overnight preculture was used for initiating the batch phase, followed by a fed-batch phase and a subsequent induction phase (addition of 0.1 mM IPTG). pO_2_ and temperature were controlled throughout cultivation at 30 % and 35 °C, respectively. The pH during batch and non-induced fed-batch was kept constant at 7.2. During the induced fed-batch, the pH was either kept constant at 7.2 (Run1), or ramped from 7.2 to 5.7 (Run2) or from 7.2 to 8.7 (Run3) as shown in Electronic Supplementary Material (ESM) Table S1. Samples were taken every hour throughout the induction phase for offline determination of cell death by FCM and for chromatogram fingerprinting.

### Flow cytometry

FCM was carried out according to Langemann et al. [11]. In short, cultivation broth was diluted to stay within the linear range of the detector of the FCM device (CyFlow® Cube 8 flow cytometer, Partec, Münster, Germany). After addition of the fluorescent dyes RH414 (abs./em. 532/760 nm, staining of all plasma membranes) and DiBAC4(3) (abs./em. 493/516 nm, membrane potential-sensitive dye for assessment of viability), data were collected using the software CyView Cube 15 and analyzed with the software FCS Express V4 (DeNovo Software, Los Angeles, CA, USA). The error in FCM measurements was always below 5 %.

### Multivariate data analysis

#### Data acquisition

A modular HPLC setup (PATfinder™) with an auto-sampler (Optimas), pump module (Azura P 6.1 L), a multi-wavelength UV detector (Azura MWD 2.1 L) and a monolithic CIMac QA column (0.1 mL) was purchased from BIA separations (Ljubljana, Slovenia). Cell-free culture supernatants were diluted 1:5 with loading buffer (50 mM Tris-HCl, pH 8; AEX-A) to avoid deviations in the background matrix. Then, 50 μL of the prepared samples were loaded onto the column and bound proteins and nucleic acids were eluted using a linear gradient with 50 mM Tris-HCl + 1 M NaCl, pH 8 (AEX-B). Summarizing, column equilibration was done for 20 column volumes (CVs) with AEX-A, followed by sample injection, 10 CVs post-injection wash with AEX-A and elution with a linear gradient of AEX-B for 20 CVs. The time required for acquiring chromatographic data of one sample was shorter than 5 min. The column was cleaned with 1 M NaOH + 2 M NaCl for 10 CV after each sample to avoid carry-over. The flow velocity was kept constant at 283 cm/h. UV chromatographic data at 260 nm were recorded to follow release of nucleic acids. The chromatographic data were logged at a frequency of 5 Hz.

#### Data preprocessing

UV chromatographic raw data are usually attributed with shifts along the retention time and the baseline, which both strongly influence further data analysis. In order to overcome these shortcomings, peak alignment and baseline correction were done using icoshift [12] and first-order derivative, respectively. The preprocessed chromatographic UV data were then arranged as chromatogram fingerprints for further data analysis. Chromatogram fingerprints can be defined as a set of preprocessed overlaid chromatographic data which can be compared to identify and explain phenomena in a process. In our study, mean-centering and scaling of the UV chromatograms at 260 nm as fingerprints were done prior to performing PCA using SIMCA (Umetrics, Umea, Sweden).

#### Pattern recognition using PCA

Principal component analysis is a widely used exploratory technique which helps in decomposing huge datasets such as the matrix X of the chromatogram fingerprints. The matrix X is represented after PCA by few latent variables, called principal components (PCs). The transformation of X to PCs results in different attributes that are associated with X, called scores and loadings. The loadings of the PCs provide an overview of the variability in the X matrix. In general, the first PCs explain most of the variance in X. The loadings explain at which retention time the variance in the chromatographic data was significant. For example, the first loading would show at which retention time the variance in the chromatographic data was high. An overview of scores plotted along different PCs reveals groupings/clusters explaining similar trends and/or deviations between different samples in X.

Although the PCA score plots can be interpreted to monitor bioprocesses with respect to various PCs [13], multi-dimensional analysis of scores and loadings is cumbersome. Therefore, we used a univariate statistic (Hotelling’s T2) from the PCA model to follow deviations from pre-defined operating conditions in the *E. coli* bioprocesses [13].

### Software

Preprocessing of chromatographic data was done in MATLAB R2015a v8.5 (Mathworks, MA, USA). Pattern recognition using PCA was done in SIMCA v13.0 (Umetrics, Umea, Sweden).

## Results

### Data acquisition and preprocessing

UV chromatographic data were acquired using a UV-VIS detector at 260 nm. After preprocessing, UV chromatographic data at 260 nm were arranged as chromatogram fingerprints as shown in ESM Fig. S1.

### Pattern recognition using PCA

**Fig. 1:**
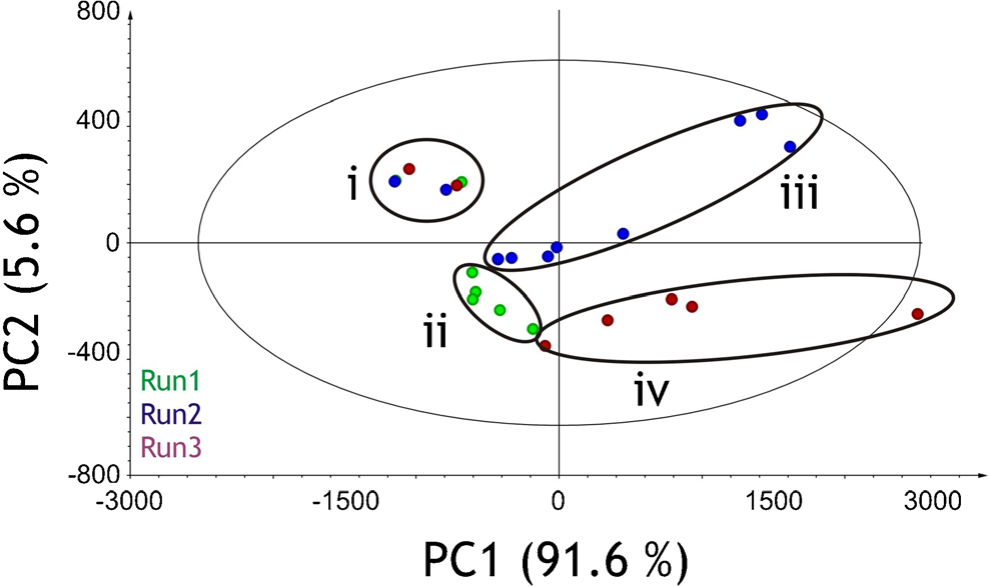
PCA score plot depicting the variation in chromatogram fingerprints at 260 nm of the three different *E. coli* bioprocesses. Cluster *i*, samples prior to lysis trigger; cluster *ii* with *green circles*, scores from *Run1*; cluster *iii* with *blue circles*, scores from *Run2*; and cluster *iv* with *red circles*, scores from *Run3*. Goodness of fit (*R* ^2^): 0.997; goodness of prediction (*Q* ^2^): 0.994

A PCA model for UV chromatograms at 260 nm as fingerprints from the three different *E. coli* bioprocesses was established. We achieved a goodness of fit (*R* ^2^) of 0.997 and goodness of prediction (*Q* ^2^) of 0.994 with seven PCs from the PCA model. The PCA score plot for the UV chromatograms as fingerprints of the different experiments was used to identify clusters (similarities) and patterns (variances) along different PCs (Fig. 1). The significance level is indicated with an eclipse surrounding the scores as 95 % confidence limit. It is interesting to note that the variance in chromatogram fingerprints during the initial phase of the different cultivations prior to intended pH ramps were similar. In other words, the impurity release pattern prior to pH ramps was similar in each bioprocess. From the score plots, we can speculate that after start of the pH ramps the impurity pattern in each experiment changed. This can be seen in the deviation from initial conditions in cluster i and other groupings where lysis was triggered with pH ramps (clusters ii, iii, and iv).

**Fig. 2:**
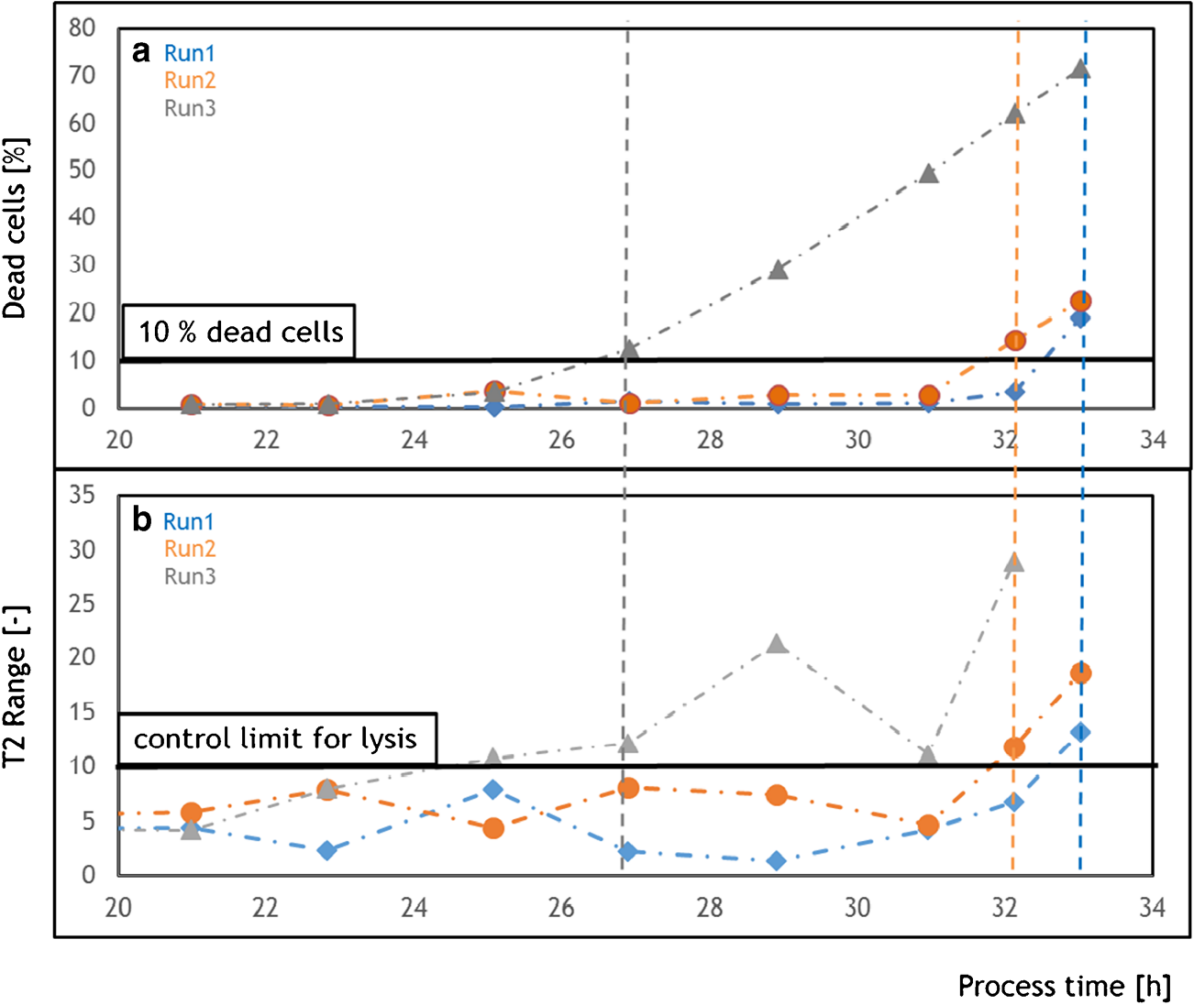
FCM offline data and the Hotelling’s T2 statistics for the three *E. coli* bioprocesses. **a** FCM data depicting cell death over process time; **b** Hotelling’s T2 statistics from the PCA model developed with chromatogram fingerprints at 260 nm over process time. *Blue diamonds*, *Run1*; *orange circles*, *Run2*; and,*gray triangles*, *Run3. Dotted line*, process deviation from control limit

Hotelling’s T2 statistic explains how well a model explains the variances in the data with respect to PCs [13, 14]. During initial stages of bioprocess development, the control limits for the univariate Hotelling’s T2 statistics need to be defined. Control limits can be established based on the T2 statistic value of the samples at the beginning of the cultivation where the impurity release patterns are similar. We defined the control limits based on the T2 statistic of the initial phase of the process under optimal conditions and found the control limit in the T2 range of 10 (Fig. 2). Once control limits are established, process deviations can be monitored and unwanted loss in product quality or quantity can be avoided.

The FCM offline data and the Hotelling’s T2 statistics, calculated from the PCA model, are shown in Fig. 2. The Hotelling’s T2 statistics showed clear deviations from the control limit in each bioprocess. In fact, these deviations happened at the same time when cell death increased (indicated by thin dotted lines in Fig. 2). Apparently, cells started to die at different time points due to the pH ramps. Cell death resulted in lysis and thus in the release of impurities (nucleic acids), which we were able to reliably detect by UV chromatograms as fingerprints and combined data analysis. Based thereon, the time point at which the bioprocess started to deviate from normal operating condition was defined as the optimal time point of harvest. With the implementation of this novel monitoring toolbox, online detection of physiological events in the bioreactor is possible, and cumbersome offline analytics along bioprocesses is minimized.

## Discussion

We implemented a novel toolbox comprising UV chromatogram as fingerprints and chemometric techniques to monitor cell death in *E. coli* bioprocesses and to define the optimal time point of harvest.

The novelty of this approach is the use of whole UV chromatogram as fingerprints, rather than single chromatogram peaks, in combination with multivariate data analysis (MVDA) tools for monitoring of bioprocesses. Chromatogram fingerprinting approaches have been only used in chemical formulations and in some downstream bioprocesses so far, but for the first time we showed the applicability of this technique in upstream process monitoring. We envision the implementation of this toolbox for monitoring different unit operations in a bioprocess, such as bioreactor cultivations, harvesting, and product purification, which will facilitate continuous bioprocessing and process development.

## Electronic supplementary material

Below is the link to the electronic supplementary material.

ESM 1 (PDF 205 kb)
