# Supplementary material for: A novel toolbox for E. coli lysis monitoring
Source: Anal Bioanal Chem. 2016 Sep 2;409(3):667–71. doi: 10.1007/s00216-016-9907-z (PMC5233743; doi:10.1007/s00216-016-9907-z)
Supplement: Supplementary file 1 — (PDF 205 kb) [file 216_2016_9907_MOESM1_ESM.pdf]

Analytical and Bioanalytical Chemistry

Electronic Supplementary Material

A novel toolbox for *E. coli* lysis monitoring

Vignesh Rajamanickam, David Wurm, Christoph Slouka, Christoph Herwig, Oliver Spadiut

Table S1 Overview of process parameters to intentionally trigger cell lysis

Experiment ID	Changed parameter	Mode of change	Detail
Run1	-	-	pH constant at 7.2
Run2	pH	Ramp	pH 7.2-5.7 in 24h
Run3			pH 7.2-8.7 in 24h

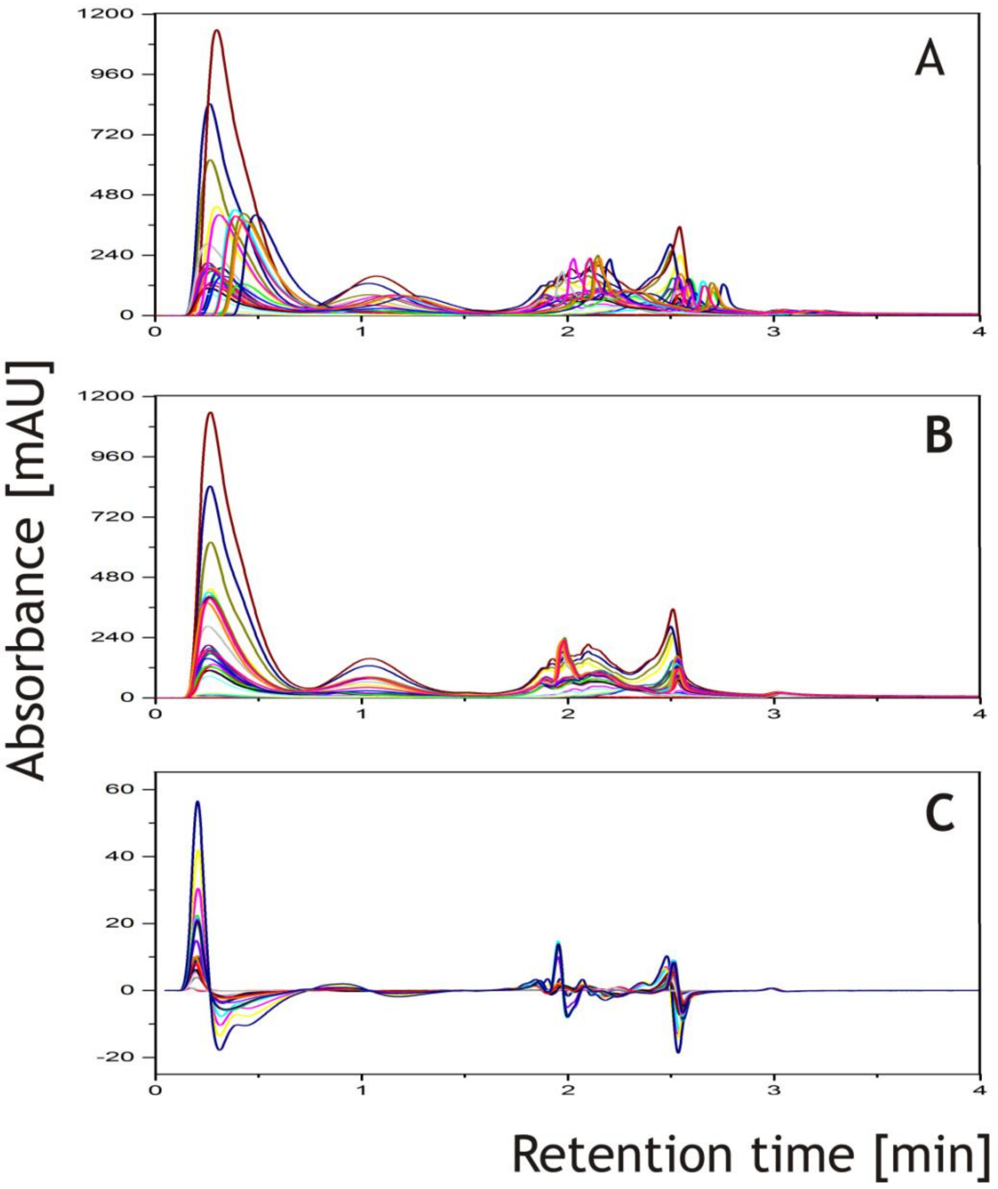

Fig. S1 Preprocessing of UV chromatographic data at 260 nm for chromatogram fingerprinting; 2A, chromatographic raw data from UV detector; 2B, aligned chromatographic data to avoid shifts in retention time; 2C, first order derivative of aligned chromatographic data for baseline correction
